# Effect of conjugated linoleic acid on blood pressure: a meta-analysis of randomized, double-blind placebo-controlled trials

**DOI:** 10.1186/s12944-015-0010-9

**Published:** 2015-02-18

**Authors:** Jing Yang, Hai-Peng Wang, Ling-Mei Zhou, Li Zhou, Tan Chen, Li-Qiang Qin

**Affiliations:** Department of Nutrition and Food Hygiene, School of Public Health, Soochow University, 199 Renai Road, Dushu Lake Higher Education Town, Suzhou, 215123 China; Department of Clinical Nutrition, The First Affiliated Hospital of Soochow University, 188 Shizi Street, Suzhou, 215006 China; Department of Cardiology, The First Affiliated Hospital of Soochow University, 188 Shizi Street, Suzhou, 215006 China

**Keywords:** Conjugated linoleic acid, Dairy products, Blood pressure, Meta-analysis

## Abstract

**Background:**

Numerous studies on animals evidenced that conjugated linoleic acid (CLA) could decrease blood pressure (BP) in several rat models. However, such beneficial effect is not completely supported by studies on humans.

**Methods:**

We searched the Pubmed, Cochrane Library, and the ClinicalTrials.gov databases for relevant randomized, double-blind placebo-controlled trials up to August 2014 to perform a meta-analysis. A random-effects model was used to calculate the combined treatment effects.

**Results:**

Eight studies with nine trials, which involved 638 participants with CLA supplementation ranging from 2.0 g/day to 6.8 g/day, were included in this meta-analysis. Compared with placebo, the pooled estimate of change was −0.03 mm Hg (95% CI: −2.29, 2.24, P = 0.98) and 0.69 mm Hg (95% CI: −1.41, 2.80, P = 0.52) in systolic and diastolic BPs, respectively. No significant heterogeneity across studies for systolic BP; however, substantial heterogeneity for diastolic BP was identified. Publication bias was not found for both systolic and diastolic BPs.

**Conclusion:**

The findings of this meta-analysis did not support the overall favorable effect of CLA supplementation on BP regulation.

## Introduction

Conjugated linoleic acid (CLA) is a mixture of positional and geometric isomers of linoleic acid, an 18-carbon polyunsaturated fatty acid. CLA isomers are naturally occurring fatty acids identified in ruminant animals. The most commonly studied CLA isomers are the cis (c)9, trans (t)11 and the t10, c12-CLA isomers [1]. In general, commercial CLA supplements usually equally contain these two active isomers, whereas, CLA in dairy products consists over 90% of c9, t11-CLA isomer [2].

Trans fatty acid may adversely influence human health; yet, evidence has shown that CLA exerts many beneficial effects, such as anti-obesity, anti-diabetic and anti-inflammatory properties [3]. Of note, CLA was also found to lower blood pressure (BP) among various rat models [4-8]. A possible mechanism by which CLA might influence BP could be through endothelial function, NO production and eicosanoids production [8,9]. In this regard, CLA may also influence BP of humans. Several human studies demonstrated that CLA supplementation can lower BP [10-13]; however, others showed no significant BP-lowering effect [14-21]. These inconsistent findings may result from variation in sample size, study population, or study quality. Thus, the present study aimed to systematically examine the effect of CLA supplementation on BP by conducting a meta-analysis of randomized controlled trials (RCTs) designed by double-blind and placebo.

## Materials and methods

### Search strategy

We followed the Preferred Reporting Items for Systematic Reviews and Meta-analysis (PRISMA) guidelines in the report of this meta-analysis [22]. We searched Pubmed, Cochrane library and the ClinicalTrials.gov databases through August 2014 for relevant studies, using terms of “conjugated linoleic acid” or “CLA” in combination with “blood pressure” or “hypertension”. No restriction was imposed. In addition, we carried out a manual search using reference lists of original articles and recent reviews.

### Study selection

Studies were included if they 1) were randomized, double-blind placebo-controlled trials in adults (Age ≥ 18 years old); 2) CLA was the only active intervention in treatment group; 3) had intervention duration ≥ 4 weeks; 4) had a control or a comparison group; 5) included the net changes of systolic and/or diastolic blood pressure (SBP/DBP) and their corresponding standard deviation (SD) or available data to calculate these values.

### Data extraction

The data were extracted independently by two researchers in duplicate (J Yang, HP Wang) according to the described selection criteria using an electronic form. Disagreement was resolved by discussion with the third research (LQ Qin). The following data were extracted from study: first author’s name, publication year, study design, intervention method, study period, sample size, daily dose of CLA (c9, t11 and t10, c12 isomers). We also extracted the following participant characteristics: gender, mean age, body mass index (BMI), baseline SBP/DBP and their changes of each study. Study quality was assessed by a modified Jadad Scale [23], where total score ranges 0 to 7 points based on their description of randomization, concealment of allocation, double blinding, withdrawn or drop-outs explanation.

### Statistical methods

The net changes were calculated as the difference between the baseline and final values of BP. If only SD for the baseline and final values were provided, SD for the net changes were imputed according to the method of Follmann using a correlation coefficient of 0.5 [24]. Overall effect size was expressed as weighted mean difference (WMD) with 95% confidence interval (CI) using Stata11 (StataCorp, College Station, TX, USA). The heterogeneity between the studies was tested using the Q test at the P < 0.10 level of significance and quantified by the *I*^2^statistic, which describes the inconsistency across studies [25]. In general, the random-effects model was used in the presence of significant heterogeneity. In fact, heterogeneity always exists in varying degrees. Thus, results from random effects model, which would be more conservative (and hence more appropriate), were presented in our meta-analysis. We did not conduct subgroup analysis because of the small number of trials. Rather, we performed a sensitivity analysis, in which a single trial was omitted each time and the effect size was recalculated to investigate its influence on the overall effect size. Furthermore, we conducted meta-regression analysis to explore possible sources of heterogeneity across studies. To minimize the likelihood of false-positive results, we carefully selected a few covariates, including CLA dose, intervention duration and baseline BP. Potential publication bias was assessed using the Begg’s funnel plots and Egger’s regression test [26]. A P <0.05 was considered statistically significant, except where otherwise specified.

## Results

### Search results

**Figure 1:**
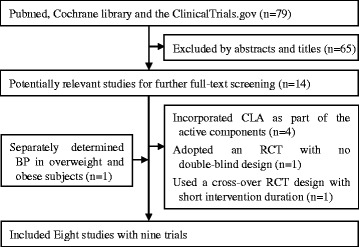
**Flow chart of study selection.**

A flow chart of literature search and study selection is presented in Figure 1. A total of 79 potential relevant articles and 14 trials were retrieved for further assessment. Six of these studies, however, were excluded from the analysis because four of them [11,13,27,28] incorporated CLA as part of the active components in treatment group, one adopted an RCT with no double-blind design [19], and the other one used a cross-over RCT design with short intervention duration (3 weeks) [20]. Meanwhile, one study separately determined BP in overweight and obese subjects and was considered as two trials [16]. Finally, eight studies with nine trials were included in this meta-analysis [10,12,14-18,21].

### Study characteristics

The characteristics of the included studies are presents in Table 1. These studies were published from 2000 to 2013, in which five were conducted in Europe, two in Iran, and one in Japan. Sample sizes varied from 38 to 346 with a sum of 318 in the CLA groups and 320 in the control groups. In five studies, both men and women were included as participants, whereas the remaining studies included only men. Except for the study of Raff that involved apparently healthy adults [14], the others included overweight and obese adults (BMI ≥ 25 kg/m^2^). No study specified the hypertension status of participants, which the majority were normotensive as indicated by mean BP levels at baseline. Two trials were conducted in patients with rheumatoid arthritis and type 2 diabetes mellitus, respectively [17,21]. Dose of CLA varied from 2 g/day to 6.8 g/day, with a median of 3 g/d. All studies supplemented CLA with a mixture of isomers, and 50:50 isomer blends were used except in the study of Sluijs [18]. Only one trial had full Jadad score (=7). Tree trials had relatively low Jadad score (=4). On the other hand, two studies performed intention-to-treat analysis. Intervention duration lasted from 5 weeks to 24 weeks and 5 studies for 12 weeks.

**Table 1:** **Characteristic of the trials and participants in this meta-analysis**

**Study**	**Country**	**No. of CLA/Control**	**Age (Year)**	**Male (%)**	**BMI (kg/m2)**	**Baseline SBP/DBP (mm Hg)**	**CLA amount (g/day)** ^***c***^	**Placebo**	**Duration (Weeks)**	**Jadad scores**
Berven 2000	Norway	25/22	47.1	63.8	29.7	139.7/85.7	2.65 (1.33)	olive oil	12	5
Raff 2006	Denmark	18/20	25.9	100	22.3	119.5/61.6	4.7 (2.35)	Control diet	5	4
Taylor 2006	UK	21/19	46	100	33.0	124.9/82.4	3.2 (1.62)	olive oil	12	6
Iwata 2007	Japan	20/20	41.5	100	28.0	127.5/77.1	6.8 (3.4)	safflower oil	12	6
Laso 2007	Spain	10/11^*a*^ 10/13^*b*^	53.9	75.0	27.6^*a*^ 33.1^*b*^	145.5/82.5^*a*^ 148.5/86.0^*b*^	3 (1.5)	Skimmed milk	12	4
Aryaeian 2008	Iran	22/22	47.1	13.6	27.8	119.8/73.5	2 (1)	oleic sunflower	12	5
Sluijs 2010	Netherland	173/173	58.4	48.3	27.9	127.4/76.1	3.1 (0.6)	palm oil	24	7
Shadman 2013	Iran	19/20	45.3	46.2	27.2	122.6/81.2	3 (1.5)	Soybean oil	8	4

^*a*^Overweight participants; ^*b*^Obese participants; ^*c*^Amount of t10, c12-CLA is presented in parentheses.

### Effect of CLA on BP

**Figure 2:**
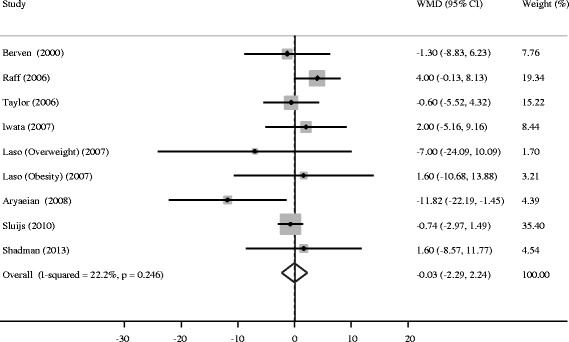
**Meta-analysis of the effect of conjugated linoleic acid supplementation on systolic blood pressure as compared with control.** WMD, weighted mean difference.

**Figure 3:**
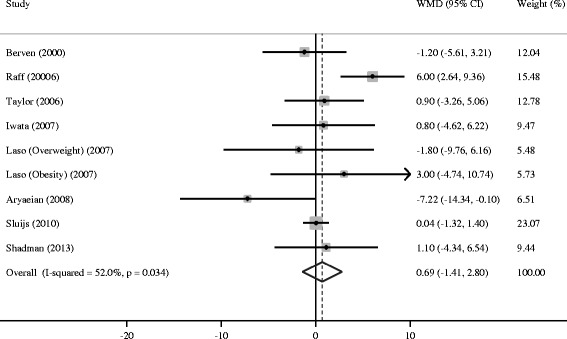
**Meta-analysis of the effect of conjugated linoleic acid supplementation on diastolic blood pressure as compared with control.** WMD, weighted mean difference.

Compared with placebo, CLA supplementation was associated with an average net change ranging from −11.82 to 4.00 mm Hg for SBP and from −7.22 to 6.00 mm Hg for DBP. SBP and DBP reductions were statistically significant in only one trial [17], whereas the other trial observed a significant DBP increase [14]. The pooled estimate of change in SBP was −0.03 mm Hg (95% CI: −2.29, 2.24; P = 0.98), without significant heterogeneity (*I*^2^ = 22.2%, P = 0.25) (Figure 2). Meanwhile, the pooled estimate of change in DBP was 0.69 mm Hg (95% CI: −1.41, 2.80; P = 0.52), with substantial heterogeneity across trials (*I*^2^ = 52.0%, P = 0.03) (Figure 3). Thus, there was no overall effect of CLA supplementation on both SBP and DBP. Such supplementation even elevated DBP without significance. Neither Begg’s test nor Egger’s test provided evidence of publication bias (all P > 0.05).

### Sensitivity analysis

Additional analyses examining the influence of an individual trial on the overall effect size by omitting one trial in each turn yielded a range from 0.22 mm Hg (95% CI: −1.45, 1.90) to–0.27 mm Hg (95% CI:-2.83, 2.29) and from 0.92 mm Hg (95% CI:-1.45, 3.28) to-0.06 mm Hg (95% CI:-1.20, 1.08) for SBP and DBP, respectively. None of the individual studies appeared to have appreciable impacts on the overall combined effect sizes.

### Meta-regression analyses

Meta-regression analysis was subsequently conducted to assess whether BP change is related to CLA dose, intervention duration, or baseline BP levels. The results of the analysis revealed that none of these covariates had significant influences on the overall effect sizes (Table 2). However, a trend toward greater reductions in SBP among subjects with higher CLA dose (r = −1.78, P =0.07) was observed.

**Table 2:** **Characteristics associated with net change in blood pressure: univariate meta-analysis analysis**

	**Systolic BP**		**Diastolic BP**	
	**Coefficient (95% CI)**	**P**	**Coefficient (95% CI)**	**P**
Baseline BP	−0.11 (−0.53, 0.30)	0.54	−0.23 (−0.61, 0.14)	0.13
Dose	−1.78 (−3.76, 0.20)	0.07	−1.32 (−3.20, 0.56)	0.14
Duration	−0.16 (−0.53, 0.20)	0.32	−0.19 (−0.61, 0.23)	0.32

## Discussion

Hypertension significantly contributes to the morbidity and mortality associated with cardiovascular disease. In this event, hypertension must necessarily be prevented. Nevertheless, this meta-analysis of randomized, double-blind, placebo-controlled trials found that supplemental CLA does not affect human BP regulation.

In particular, the results of this analysis contradict those of the animal studies, which consistently reported the BP lowering effects of CLA [4-8]. Such discrepancy between human and animal studies may because of the characteristics of observational subjects as animal models already have established hypertension. In the studies including in this meta-analysis, most participants were generally normotensive, in which further decreasing BP was not probable. To surprise, the study of Laso with a relatively higher BP (SBP > 130 mmHg or DBP > 85 mmHg) also did not observe BP change for supplementation with 3 g of CLA for 12 weeks [16]. Two human trials reported BP lowering effects of CLA. Herrera discovered that CLA combined with calcium could reduce pregnancy-induced hypertension [11]. Zhao identified that CLA supplementation could enhance the antihypertensive effects of Ramipril among stage 1 hypertension patients [13]. The participants in these two studies were hypertensive, further suggesting the importance of baseline BP for CLA effect. These two studies were excluded in the current meta-analysis because they did not satisfy the selection criteria.

Obesity is a major factor that promotes hypertension. Participants were mostly overweight or obese adults in this meta-analysis. An animal study demonstrated that CLA could reduce the number of large adipocytes, thereby contributing to obesity-related hypertension [7]. In study of Laso, the participants were divided into two groups according to their BMI. Surprisingly, CLA supplementation significantly decreased fat mass in overweight participants, but not in obese [16]. As described above, no change of BP was observed in this population. In fact, elevated BP after CLA supplementation was observed in the study of Raff, where the participants had relatively low BMI [14].

CLA dose is another relevant factor of BP regulation. Meta-regression analyses found a trend toward greater reduction in SBP among participants with higher CLA dose. In most animal experiments, the daily intake of 0.55 to 1% of CLA is attributed to nearly all of the beneficial effects of CLA [29]. Plasma CLA concentration can reach 200 mM in the rodent animals followed by dietary intakes of 0.5% CLA [30]. If plasma CLA concentration was taken as a reference parameter, the daily intake of 3.2 g of CLA in humans must attain the comparable level [31]. Most of the studies in this meta-analysis reached this particular CLA dose. Hence, beneficial effect was expected to be observed in humans. Contrarily, no BP lowering effect was identified in these studies. Thus, apart from CLA dose, different mixture of isomers must also be considered. Divergent effects of c9, t11 and t10, c12 isomers on BP were observed in animal studies when these two isomers were separately used [4,7]. Interestingly, t10, c12, not c9, t11 isomer, significantly suppressed the development of hypertension [4]. The different effects of c9, t11 and t10, c12 isomers were also reported on blood lipid and insulin resistance in human research [32,33]. However, this meta-analysis did not involve human studies that observed the separated isomer of CLA on BP. In study of Iwata, the contents of t10, c12 and c9, t11 isomers were both 3.4 g, which was the highest measure among all studies. Although BP was significantly deceased at 12 weeks than at the baseline, BP was simultaneously decreased in the control group, resulting in no beneficial effect of CLA [12].

Several limitations of this meta-analysis must be acknowledged. First, only nine randomized trials were included; thus, subgroup analyses were planned, but not performed. Such analyses stratified by location, intervention duration, or study design might be informative, yet they would have increased the risk of type I errors. Second, most studies included in this meta-analysis were not primarily designed to investigate the CLA effect on BP. Thus, factors related with BP were maybe vague, such as intentional lifestyle or behavioral change, method to measure BP. Third, food intake was not controlled in several studies, and the participants may possibly consumed extra CLA from diet source. However, the daily intake of CLA in ruminant products was estimated 152 and 212 mg in women and men, respectively [34]. Theses measures can be neglected in relatively higher CLA supplementation. Finally, substantial heterogeneity across trials in DBP was determined. In this meta-analysis, random effects model was used to estimate the overall effect size.

Our study had some strengths. Because individual studies had insufficient statistical power, our meta-analysis enhanced the power to detect a possible association and provided more reliable estimates. All included studies were randomized, double-blind, placebo-controlled trials, which minimized biases and suggested a high internal validity. In addition, results of sensitivity analyses supported the robustness of the findings. Finally, publication bias, the selective reporting of studies featuring positive or extreme results, may result in overestimation of relationship between CLA and BP. However, no publication bias was found in this meta-analysis.

On the basis of the current evidence, the findings of this work do not support the overall favorable effect of CLA supplementation on BP regulation. Whether the effects of CLA on BP are related to baseline BP, obese status, and intervention dose or duration is yet to be determined. Further studies must be accumulated for subgroup analysis according to the above characteristics and study design.
